# The impact of environmental factors, sowing time, and soil conditions on the growth, yield, and resilience of *Fagopyrum tataricum* and *Fagopyrum esculentum* in Egyptian agroecosystems

**DOI:** 10.1371/journal.pone.0344039

**Published:** 2026-05-14

**Authors:** Mohamed M. Hassona, Nahla M. Morsy, Ahmed M. S. Hussein, Hala A. Abd El-Aal

**Affiliations:** 1 Qur’anic Botanic Garden, Hamad bin Khalifa University (HBKU), Doha, Qatar; 2 Department of Sustainable Development of Environment and Its Projects Management, Environmental Studies & Research Institute (ESRI), University of Sadat City (USC), Sadat City, Menoufia, Egypt; 3 Food Technology Department, National Research Center, Cairo, Egypt; Baotou Medical College, CHINA

## Abstract

**Background:**

Buckwheat (*Fagopyrum* spp.) is a gluten-free pseudocereal with high nutritional and bioactive value, offering the potential to diversify Egyptian cropping systems under semi-arid conditions. However, genotype × environment × management interactions remain poorly characterized for common (*F. esculentum*) and Tartary (*F. tataricum*) buckwheat in Egypt.

**Methods:**

Field experiments were conducted over two growing seasons (2018/19–2019/20) at two contrasting northern Egyptian sites: the Belbies city site (clay–sandy, irrigated Nile Delta) and the Sadat city site (sandy–loamy, desert fringe). A three‐factor factorial randomized complete block design (RCBD) with three replicates per site was used to evaluate the species (Common vs. Tartary), location (Belbies vs. Sadat), and sowing date (mid-November, mid-January, and mid-March). Vegetative traits (height, branch number, internode number, leaf number, fresh biomass) and productivity parameters (seed count, seed weight, yield) were measured on ten plants and in three 1 m^2^ quadrats per plot. Two‐step ANOVA (species‐specific two‐way followed by combined three‐way) and LSD mean separation were applied (α = 0.05).

**Results:**

Three‐way interactions among species, location, and sowing date were highly significant for all traits (p < 0.001), explaining 18–32% of the variance. Tartary buckwheat outperformed common buckwheat across both sites and seasons, exhibiting greater vegetative vigor and reproductive resilience. At Belbies, mid-March sowing of *F. tataricum* resulted in the highest grain yields (947 ± 22 kg ha ⁻ ¹ in 2018/19; 997 ± 25 kg ha ⁻ ¹ in 2019/20), whereas common buckwheat yields peaked at mid-November sowing (558 ± 19 and 491 ± 18 kg ha ⁻ ¹, respectively). At Sadat, yields were lower for both species but remained consistently greater for *F. tataricum* on all sowing dates. Compared with *F. tataricum*, *F. esculentum* performed worse under later sowing and desert-fringe conditions.

**Conclusions:**

Tartary buckwheat presented greater growth and yield than did common buckwheat across the two sites and sowing windows tested. Within Belbies (clay–sandy), mid-March sowing resulted in the highest *F. tataricum* yields; at Sadat (sandy–loamy), yields were lower across dates. These results indicate that *F. tataricum* is a promising candidate for Egyptian agroecosystems, with location- and date-specific performance.

## Introduction

Agriculture in Egypt faces persistent challenges, including water and land scarcity, overreliance on the Nile for irrigation, and limited arable land, all of which are exacerbated by rapid population growth and climate change, threatening the country’s ability to sustainably meet rising food demand and ensure food security [1–3,4,5,6]. These agricultural constraints contribute to malnutrition, as the food system struggles to provide a healthy, diverse diet, resulting in a “double burden” of undernutrition and overnutrition; this burden is manifested by high rates of child stunting, micronutrient deficiencies, and increasing obesity, placing a significant economic and health burden on the nation [7,6]. Within this broader context, diversifying crop production emerges as a critical priority. One promising candidate is buckwheat (genus *Fagopyrum*), specifically the two major cultivated species: common buckwheat (*Fagopyrum esculentum* Moench.) and Tartary buckwheat (*Fagopyrum tataricum* Gaertn.). These species offer unique potential for crop diversification in Egyptian agroecosystems, necessitating a thorough investigation into their adaptability, productivity, and resilience under local environmental conditions. However, Buckwheat, which belongs to the Polygonaceae family, is recognized as a pseudocereal crop characterized by triangular seeds and broad, heart-shaped leaves. It originated in the Himalayan region, particularly western China, and has since spread globally through historical trade and migration routes [8–13]. Its global cultivation extends across Asia and Europe and has adapted broadly to temperate and semiarid climates owing to its intrinsic resilience to various environmental stresses, including drought, cold temperatures, and low soil fertility [10,13–15]. On the other hand, Buckwheat grains are distinguished nutritionally by their high protein content, essential amino acids, dietary fiber, resistant starch, vitamins (especially B vitamins), and minerals such as iron, magnesium, and selenium [16–21]. Owing to their notable antioxidant, anti-inflammatory, and cardiovascular health benefits, unique bioactive compounds such as flavonoids (specifically rutin and quercetin) are valuable [16,22,23]. Furthermore, buckwheat is gluten-free, making it an important dietary staple for individuals with gluten sensitivity and celiac disease [22,20]. Thus, the introduction of buckwheat into Egypt’s agricultural systems aligns strategically with national goals aimed at diversifying crop [1] portfolios, enhancing nutritional security, and improving climate resilience. However, successful integration necessitates an extensive evaluation of how different environmental parameters (such as temperature, water availability, and soil conditions) influence the vegetative growth and productivity of buckwheat species.

The vegetative responses of buckwheat species differ significantly under various environmental stresses. Compared with common buckwheat, Tartary buckwheat typically presents superior growth traits, producing greater node and branch numbers and maintaining vegetative productivity under stress conditions [24–26]. High-temperature exposure can alter leaf production dynamics; notably, *F. tataricum* increases leaf formation as a compensatory mechanism under thermal stress, indirectly influencing plant height and branching characteristics [27]. These variations in morphological traits, such as internode elongation, result from genetic‒environmental interactions that significantly affect plant adaptability and growth performance [28,29]. However, environmental stresses, particularly water deficits, notably affect buckwheat growth. Compared with F. esculentum, *F. tataricum* is more drought *tolerant*, maintaining growth even under limited water conditions, whereas common buckwheat often has reduced leaf and branch production under similar conditions [27]. Previous studies reported stress-related advantages in *F. tataricum*. In the present study, we did not impose controlled stress treatments; our aim was to compare species performance under two Egyptian field contexts and three sowing dates [19,30–32]. Additionally, soil properties, such as texture, pH, salinity, and organic matter content, significantly influence buckwheat morphology and productivity [33,34]. Sandy soils, which are typical of Egypt’s desert margins, often have limited nutrient-holding capacity; however, amendments such as biochar significantly improve nutrient availability and water retention and consequently substantially increase buckwheat yield [8]. Conversely, soils with better native fertility, such as sandy loams, exhibit less dramatic yield responses to amendments but still benefit from enhanced nutrient dynamics and microbial activity [8,35]. Saline soils pose specific challenges by altering nutrient uptake patterns but simultaneously induce the accumulation of beneficial minerals (e.g., calcium, iron, zinc, and selenium) that enhance the nutritional profile of buckwheat [36]. Mitigation strategies using gypsum and polyacrylamide reduce soil salinity and optimize growing conditions, thus facilitating better crop performance [37]. Organic amendments such as vermicompost improve overall soil fertility, plant nutrient uptake efficiency, and stress tolerance, thus markedly improving grain nutrient composition and crop productivity [38]. However, the timing of sowing profoundly impacts buckwheat productivity. Early sowing typically enhances growth by exploiting favorable moisture and temperature conditions, whereas delayed sowing exposes plants to heat and drought stress, significantly reducing biomass and grain yield[39,40,41,42,43,44,45]. Late planting limits vegetative and reproductive traits due to increased abiotic stress during critical growth stages [46,47]. Seasonal variability, characterized by fluctuating temperatures, humidity, and wind patterns, further complicates the reproductive success and final productivity of buckwheat [48] species [49,50]. Optimizing productivity in arid and semiarid systems requires strategic agronomic practices. Techniques such as biochar application, seed bio stimulants, and mulching have proven effective in enhancing water retention, improving nutrient availability, and ultimately increasing yield and resilience in water-limited environments [51–54]. The integration of buckwheat into intercropping systems (e.g., with fenugreek) further enhances resource-use efficiency and overall crop productivity [53,54]. To interpret complex genotype‒environment‒management interactions effectively in buckwheat trials, robust statistical methodologies are necessary. Models such as three-way principal component analysis (PCA-SREG), additive main effects and multiplicative interaction (AMMI), and linear mixed models (REML) allow nuanced analysis of genotype × location × sowing time interactions, thus facilitating accurate identification of adaptive traits and optimal agronomic practices [55–62].

The rationale and objectives of this study are that evidence is limited on how sowing windows and the soil context shape the performance of two cultivated buckwheat species—common buckwheat (*Fagopyrum esculentum*) and Tartary buckwheat (*Fagopyrum tataricum*)—in Egypt. We therefore tested **2** species × 2 locations (clay–sandy Nile Delta vs. sandy–loamy desert fringe) × 3 sowing dates (mid-Nov, mid-Jan, and mid-Mar) to quantify the effects on growth and yield and to identify agronomically viable combinations for Egyptian agroecosystems.

## 2. Materials and methods

### 2.1. Experimental sites and climatic characterization

**Fig 1:**
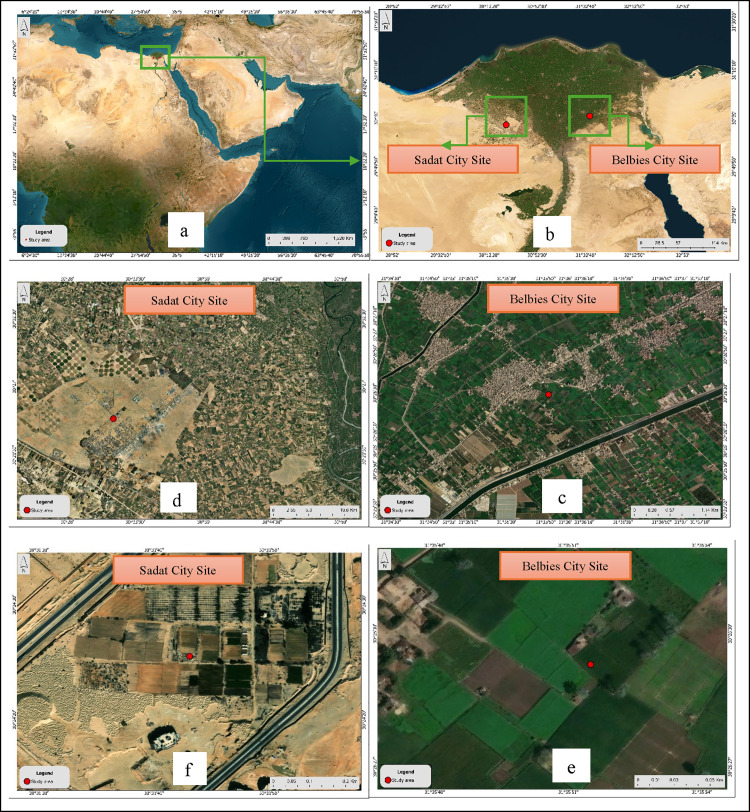
(a) Regional map showing Egypt foem a continenetal view, (b) The two cultivation sites shown from the position of the Nile Delta, north of Egypt. (c&d) Satelite view of location #1 (30.4415°N, 31.5976°E) whcih is a cultivated area near Belbies city (BCS), whereas (d) satellite view of Location #2 (30.4071°, 30.5300°E), which is located at the desert margin near Sadat city (SCS). (e) Overview of cultivation site No.1 (BCS) showing geographical and land features. (f) Close-up map of cultivation site 2 (SCS) showing the desert location where this site is used. Esri. (2024). ArcGIS Pro (Version 3.8) [Computer software]. Environment Systems Research Institute. https://www.esri.com.

Species background: *F*. *esculentum* and *F. tataricum* (Polygonaceae) are gluten-free pseudocereals that are valued for protein and flavonoids (rutin) and are widely used for food and health applications. Field trials were conducted over two growing seasons (2018/2019 and 2019/2020) at two contrasting sites in North Egypt. Site No. 1 is located at the Belbies city site (BCS) with coordinates of 30.4196° N, 31.5619° E, featuring an irrigated Nile Delta location characterized by clay–sandy soils. Site No. 2 is located at the Sadat city site (SCS), which is coordinated as 30.3594° N, 30.5327° E, as it is a desert-fringe farm at the University of Sadat city with sandy–loamy soils. The maps in Fig 1 show both sites in detail from a continental, regional, interspecific region, and close-up zoom angles sourced by a Spatial data processing, geographic analysis, and cartographic map production were conducted using ArcGIS Pro (Version 3.8) developed by Esri [63]. The workflow included preprocessing of spatial datasets, integration of vector and raster layers, execution of spatial analysis tools, and generation of thematic maps following standard cartographic principles. Advanced geoprocessing functions were applied to ensure spatial accuracy and reliable spatial representation of the studied environmental variables. Before sowing each season, composite soil samples (0–30 cm) were collected following the guidelines of the Soil, Water and Environment Research Institute (SWERI) of the Agriculture Research Center, Egypt, and analyzed for pH, EC, texture, organic matter, CaCO₃, macro- and micronutrients [64] (**Table 1**). The irrigation water was sampled at each site and characterized for pH, EC, SAR, major cations/anions, and trace metals [65] (**Table 2**). Daily meteorological data at the Belbies city site (BCS; 30.4196° N, 31.5619° E) were obtained from Meteoblue AG (Basel, Switzerland) via satellite-linked automatic weather stations [66]. Air measurements at 2 m elevation and soil temperature at 0–7 cm depth were logged continuously, yielding daily maxima, minima, and arithmetic means for air temperature (°C), relative humidity (%), wind gust (km/h), and soil temperature (°C). Precipitation (mm), snowfall amount (cm), and sunshine duration (min day ⁻ ¹) were recorded as daily totals.

**Table 1:** Presowing (0–30 cm) soil properties at the Belbies city site (BCS) and the Sadat city site (SCS) were measured before each sowing in both seasons; values represent the means of three composite samples per site per season.

*Soil Physical, Chemical, and Mechanical Properties*		Belbies City Site	Sadat City Site
pH		7.83	8.57
EC (dS/m)		1.48	1.11
SP%		25	–
Soluble anions (meq/L)	**CO3**	–	–
	**HCO3**	0.25	1.9
	**Cl**	12.1	7.4
	**SO4**	1.56	3.4
Soluble cations (meq/L)	**Ca++**	3.7	1.2
	**Mg++**	1.8	1.2
	**Na+**	8	3.3
	**K+**	0.5	0.18
	**CaCO3 (%)**	3.9	1.7
	**OM%**	1.2	0.35
Available levels of nutrients ml/kg	**N**	175	–
	**P**	183.6	5.3
	**K**	7.96	508
	**Cu**	0.75	1.43
	**Fe**	5.25	1.11
	**Mg**	2.43	20
	**Zn**	2	2.75
Mechanical analysis %	**Sand**	83	83
	**Silt**	15.5	10
	**Clay**	1.2	7
	**Texture Grade**	Clay sandy	Sandy loamy

**Table 2:** The irrigation water quality at BCS and SCS was measured at sowing for each season, with mean values (± SDs) of three replicates.

Parameter		Belbies City Site	Sadat City Site
pH		7.41	7.98
EC (dS/m)		0.64	1.26
Chemical parameter	**Ca++**	1.7	4.2
	**Mg++**	1	2.6
	**Na+**	3.2	6.5
	**K+**	0.01	0.2
	**Co3**	0	0
	**HCO3**	0.21	4
	**CI**	4.7	7
	**SO4**	10.9	2.5
	**SAR**	2.76	3.5
	**TDS**	410	896
Fe	**ppm**	0.269	0.11
Zn		0.014	0.18
Mn		0.198	<0.01
Cu		0.019	<0.01

### 2.2. Experimental design

- **Design:** A **3-factor factorial RCBD** (three blocks per site) was tested:
  - **Species (2):** *F. esculentum* and *F. tataricum* (**cultivars were not an experimental factor; commercial lots used to represent species**).
  - **Location (2):** Belbies city site (BCS; clay–sandy) and Sadat city site (SCS; sandy–loamy).
  - **Sowing date (3):** mid-November, mid-January, and mid-March.

**Treatment structure and size:** 12 combinations per site; **36 plots/site/season** (plot 3.0 × 1.5 m = 4.5 m²; 0.5 m alleys). Blocks oriented to minimize soil/microclimate gradients. Across two seasons and two sites, **144 plots** were evaluated.

### 2.3. Seed source

Seeds of *Fagopyrum tataricum* (Tartary buckwheat) and *Fagopyrum esculentum* (common buckwheat) were obtained from Sustainable Seeds Company (California, USA). The seed lots were labeled commercial cultivars suitable for research purposes and were visually inspected to confirm species identity and uniformity prior to sowing. Seeds were stored in paper envelopes under ambient laboratory conditions (≈ 22 ± 2 °C; 40–50% RH) until planting. The use of two widely available species from a single commercial source allowed a controlled comparison of their field performance under contrasting Egyptian agroecosystem conditions.

### 2.4. Cultivation site soil and water characteristics

#### Belbies city site (BCS).

The soil at BCS is a clay–sandy loam, combining the rapid drainage of sand with the moisture-holding capacity of the clay. This mixed texture promotes good aeration around roots while retaining enough water to buffer short dry periods. The soil’s near-neutral pH minimizes the risk of micronutrient lock-up or aluminum toxicity, and moderate carbonate levels help stabilize pH fluctuations. The organic matter content supports active microbial communities and gradual nutrient release (Table 1). The irrigation water here is low in dissolved salts and sodium, preserving the soil structure and ensuring that essential cations (calcium, magnesium, and potassium) remain plant-available (Table 2).

#### Sadat city site (SCS).

The SCS soil is a sandy–loamy mixture with very high sand content and minimal silt or clay, resulting in rapid drainage but limited moisture and nutrient retention between irrigations. Its naturally alkaline pH and elevated bicarbonate can predispose individuals to phosphorus fixation and reduce micronutrient availability if not managed. Low organic matter means that fertility relies heavily on external amendments (Table 1). The irrigation water in the SCS has a relatively high total dissolved solids content and sodium adsorption potential, which, over time, may increase soil permeability and necessitate precise water-management strategies (Table 2).

### 2.3. Preplanting Preparation and Agronomic Management

- **Land Preparation:** At each site, fields were plowed twice (at one-week intervals) to a depth of 30–45 cm to eliminate weeds and homogenize the soil.
- **Organic Amendment & Fertilization:** One week before each sowing date, the soil was prepared by two passes of mechanical plowing to a depth of 30–45 cm with a one-week interval.

An organic amendment was applied at a rate of **10 t ha⁻¹ compost**, which was incorporated into the top 0–15 cm. The compost was derived from mixed plant residues and cattle manure, with a composition of **1.2% N, 0.5% P₂O₅, and 1.8% K₂O** on a dry-weight basis.

Additionally, **150 kg ha⁻¹ single superphosphate (15.5% P₂O₅)** was applied immediately before sowing and hand-incorporated to 0–15 cm.

No further NPK fertilizers were applied during the season.

**Fig 2:**
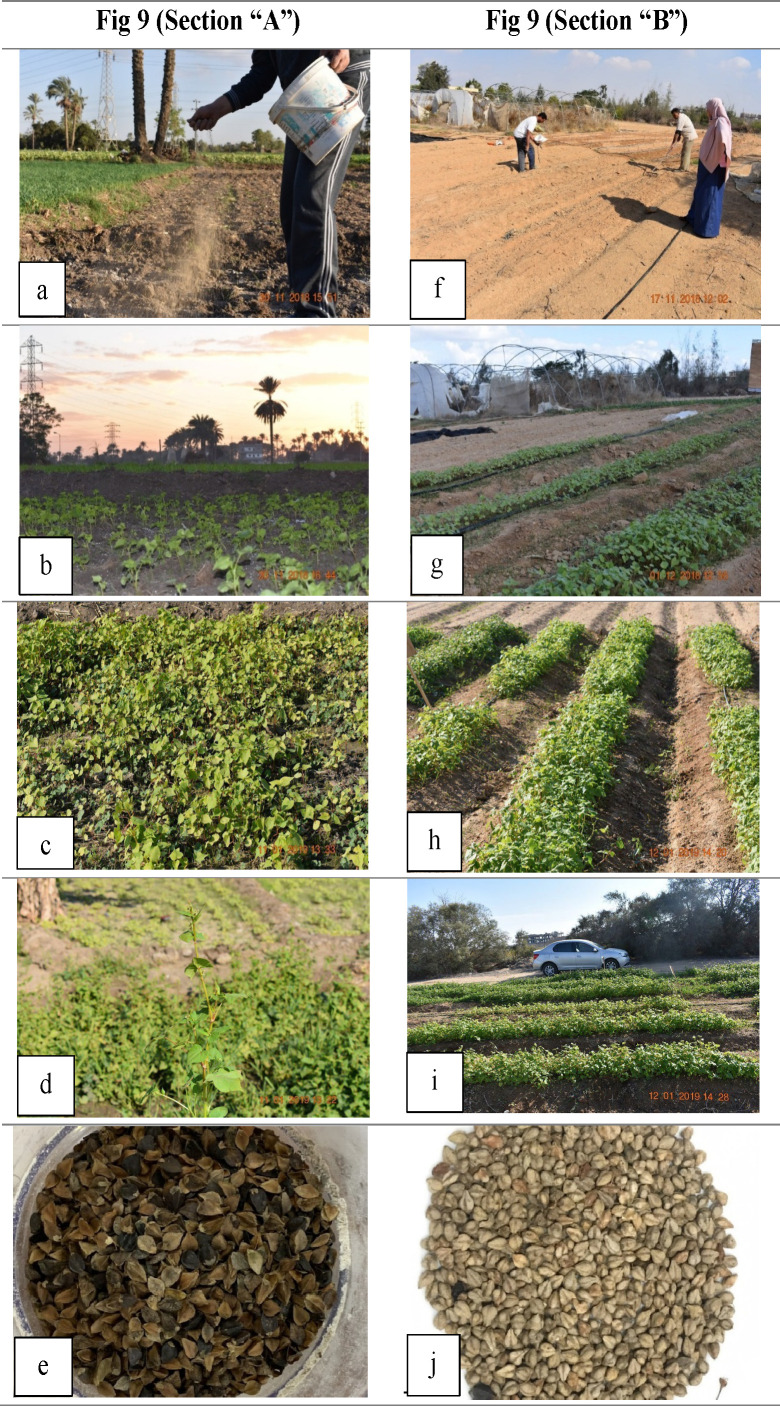
Plant heights of common and Tartary buckwheat plants across sowing dated and locations. Mean height (cm) of *Fagopyrum esculentum* and *Fagopyrum tataricum* sown at mid-November, mid-January and mid-March in Belbies city and Sadat city in 2018/19 (green) and 2019/20 (purple); bars represnt the mean ±SE of three replicates, and different letters denote significant differences among sowing-date × location treatments within each species and season (one-way ANOVA with LSD post hoc test, p<0.05).

- The soil samples presented in Table 1 were collected **before compost incorporation** at each sowing cycle to represent the baseline fertility conditions. The absence of detectable nitrogen in Table 1 reflects the preamendment status of the soil rather than the absence of fertilizer application. Postamendment nitrogen dynamics were not analyzed, as the trial focused on comparative species performance under standardized nutrient inputs.
- **Seed Preparation & Sowing:** Seeds were mixed with fine sand (2:1 v/v) and hand-broadcast at 160 seeds m ⁻ ² onto dry soil as “Afeer” (Afeer cultivation is a traditional sowing method in Egypt; Fig 2). The plots were lightly raked to ensure seed–soil contact.
- **Establishment Verification:** At 14 days after sowing (DAS), emerged seedlings were counted in three 1 m² quadrants per plot. All subsequent measurements and yield calculations were normalized to the actual plant density.

### 2.4. Irrigation and postplanting management

Immediately after sowing, each plot received a single 5-minute period of surface irrigation to ensure uniform germination. Thereafter, irrigation was applied every 10 days throughout growth, ceasing two weeks before harvest to facilitate grain ripening. The water quality parameters are provided in **Table 2**.

### 2.5. Growth and biomass assessment

All morphological and yield traits were evaluated according to the *Bioversity International Descriptor List for Buckwheat* [67,68]. For each treatment, ten plants per replicate (three replicates; n = 30) were randomly tagged at the 4-leaf stage and measured at key growth stages (BBCH 60, 71, 89) [69]. Measurements were conducted between 08:00 and 10:00 h to minimize diurnal variability. At physiological maturity (BBCH 89), vegetative growth was quantified via plants from the central 1 m^2^ of each plot to avoid edge effects. Plant height was measured from the soil surface to the main stem apex via a rigid measuring tape. Primary lateral branches and internodes were counted along the main axis, while fully expanded leaves were separated and counted to obtain the total number of leaves per plant. The aboveground biomass was harvested at ground level, placed in labeled paper bags, and weighed to the nearest 0.01 g on a calibrated digital scale to determine the fresh weight per plant. The grain yield and seed traits were determined from three 1 m^2^ quadrats per plot harvested at full maturity.

### 2.6. Productivity parameters

Productivity was assessed on the same ten plants used for vegetative measurements and in three permanently marked 1 m^2^ quadrats per plot. After physiological maturity, the heads of each of the ten sampled plants were threshed by hand and cleaned of debris, and all the seeds were counted to determine the seed count per plant. Concurrently, seeds were harvested from each quadrat, oven-dried at 65 °C to a constant weight and weighed on a precision balance; the sum of the seed mass within each quadrat was expressed as grams per square meter (g m ⁻ ^2^). The plot-level grain yield was then calculated by extrapolating the mean quadrat weight to kilograms per hectare via the formula yield (kg ha ⁻ ¹) = (mean g m ⁻ ^2^ × 10), thereby standardizing productivity comparisons across treatments.

### 2.7. Statistical analysis

A two-step ANOVA approach was adopted [70,71]:

1. **Species-specific analysis:** Separate two-way ANOVAs for each species, examining sowing time and location effects on each trait [72].
2. **Combined analysis:** A three-way factorial ANOVA (species × location × sowing time) was performed on the pooled dataset to detect higher‐order interactions.

### 2.8. Ethics statement

This study did not involve human participants or vertebrate animals. All experimental procedures, including field trials and plant material collection, were conducted in accordance with institutional, national, and international guidelines. Field experiments were carried out on agricultural research land managed by the University of Sadat City and the Belbies experimental site, with the landowners’ consent. No permits were required for the use of *Fagopyrum* species, as they are not endangered or protected.

## 3. Results

All agronomic practices, measurement timings, and environmental monitoring followed standardized protocols to ensure repeatability across seasons and locations. The procedures were documented following the Bioversity International Descriptor List for Buckwheat [67], allowing full methodological replication by future researchers. Across sections, differences among sowing dates reflect cross-sectional treatment comparisons, not temporal changes within the same stand. Three-way factorial analysis of variance revealed highly significant interactions among species, location, and sowing date for all measured traits (p < 0.001). Species × location × sowing date interactions accounted for 18–32% of the total variance, underscoring the complex, nonadditive effects of genetics, edaphic conditions, and phenology on buckwheat performance.

### 3.1. Effect of species × location × sowing date on plant height

Plant height was influenced by highly significant three-way interactions among species, location, and sowing date (p < 0.001; Table 3). At the clay‒sandy Belbies city site, *F. tataricum* consistently outgrew *F. esculentum* across all sowing dates. When sown in mid-November, *F. tataricum* reached 102.00 ± 1.22 cm in 2018/19 and 97.33 ± 1.10 cm in 2019/20, which was 8.4% lower in mid-March than in mid-November. In contrast, *F. esculentum* at the same site peaked at 79.33 ± 0.98 cm (2018/19) and 66.56 ± 1.04 cm (2019/20) under mid-November sowing and then was 14.5% lower under mid-March than under mid-November sowing. At the sandy-loamy Sadat city site, the heights of both species were lower but retained similar sowing date patterns. *F. tataricum* averaged 89.56 ± 1.15 cm (2018/19) and 81.45 ± 1.12 cm (2019/20) when sown in mid-November, which was 6.8% lower in mid-March than in mid-November. *F. esculentum* was measured at only 41.61 ± 0.87 cm and 44.67 ± 0.89 cm (mid-November), which was 38.8% lower in mid-March than in mid-November. Overall, mid-November sowing produced the tallest plants for both species at both sites, but the magnitude of the difference between mid-March and mid-November was always smaller for *F. tataricum* (6–8%) than for *F. esculentum* (14–39%). The Belbies city plants were 11–18% taller than those at Sadat for *F. tataricum* and 48–68% taller for *F. esculentum*, reflecting the integrated effects of soil texture, water availability, and the local microclimate across our uniformly managed trials (Section 2.3–2.5).

**Table 3:** Plant *height* (cm) by the interaction of *species × location × sowing date.*

Species	Planting Location	Time of Planting	2018/2019	2019/2020
*Fagopyrum esculentum*	Belbies City Site	Mid-November	79.330d	66.556e
		Mid-January	78.790de	69.444e
		Mid-March	67.790e	53.555f
	Sadat City Site	Mid-November	41.610f	44.667 g
		Mid-January	39.330f	46.556 g
		Mid-March	25.440 g	33.889 h
*Fagopyrum tataricum*	Belbies City Site	Mid-November	102.00a	97.333a
		Mid-January	97.67ab	85.889bc
		Mid-March	93.44abc	89.000b
	Sadat City Site	Mid-November	89.56bcd	81.445 cd
		Mid-January	84.00 cd	76.222d
		Mid-March	83.44 cd	77.333d
		LSD 0.05	**11.463**	**6.3703**

### 3.3. Number of branches per plant

The number of branches per plant was strongly influenced by species, location, sowing date, and their interactions (three‐way ANOVA, p < 0.001; Table 4). Overall, *Fagopyrum tataricum* exhibited markedly greater branching than *F. esculentum* across all sites × date combinations. In Belbies city, the average number of branches of *F. tataricum* sown in mid‐November was 12.56 ± 0.28 in 2018/19 and 11.22 ± 0.30 in 2019/20, which was slightly lower than the corresponding values of 11.56 ± 0.26 and 10.45 ± 0.29 under mid‐March sowing. In contrast, *F. esculentum* at the same site produced 8.33 ± 0.22 and 7.78 ± 0.24 branches in mid‐November, 7.44 ± 0.20 in mid-March and 7.56 ± 0.23 in the latest sowing. In Sadat city, branching was uniformly lower: *F. tataricum* ranged from 10.89 ± 0.25 (mid‐November) to 9.67 ± 0.23 (mid‐March) in 2018/19 and from 9.78 ± 0.27 to 9.45 ± 0.26 in 2019/20, whereas *F. esculentum* declined from 6.67 ± 0.21 to 5.33 ± 0.18 branches in the first season and from 7.11 ± 0.22 to 6.33 ± 0.19 in the second season. The least significant difference (LSD) at p = 0.05 was 0.84 branches in 2018/19 and 0.92 in 2019/20, confirming that both the location and sowing date effects were significant within each species.

**Table 4:** Number of branches per plant by the interaction of species × location × sowing date.

Species	Planting Location	Time of Planting	2018/2019	2019/2020
*Fagopyrum esculentum*	Belbies City Site	Mid-November	8.333e	7.778e
		Mid-January	8.222ef	8.000e
		Mid-March	7.444 fg	7.556ef
	Sadat City Site	Mid-November	6.667gh	7.111f
		Mid-January	6.556 h	7.000f
		Mid-March	5.333i	6.333 g
*Fagopyrum tataricum*	Belbies City Site	Mid-November	12.555a	11.222a
		Mid-January	11.889ab	10.445b
		Mid-March	11.555bc	10.445b
	Sadat City Site	Mid-November	10.889c	9.778c
		Mid-January	9.778d	9.111d
		Mid-March	9.667d	9.445 cd
		LDS 0.05	**0.8244**	**0.5786**

### 3.4. Number of internodes per plant

Analysis of variance revealed highly significant effects of species, location, sowing date, and their interactions on internode number (three‐way ANOVA, p < 0.001; Table 5). Across both seasons, *Fagopyrum tataricum* consistently produced more internodes than did *F. esculentum* under all the environmental conditions. In Belbies city, *F. tataricum* sown in mid‐November averaged 14.67 internodes in 2018/19 and 12.56 internodes in 2019/20, with slightly fewer internodes (13.78…) in mid-March than in mid-November. In contrast, *F. esculentum* at the same site developed 9.11 internodes in 2018/19 and 9.44 in 2019/20 when it was sown in mid‐November; the number of internodes was 8.22 in mid-March and 8.22 in late sowing. In Sadat city, the internode counts were lower for both species: *F. tataricum* ranged from 12.00 to 10.45 internodes in the first season (11.33–10.67 in the second), whereas *F. esculentum* declined from 7.33 to 6.56 internodes (7.78–6.33 in the second season). The least significant difference at p = 0.05 was 0.75 internodes in 2018/19 and 0.82 in 2019/20, confirming that each factor—and their three‐way interaction—significantly influenced node formation.

**Table 5:** Number of Internodes per Plant by the interaction of species × location × sowing date.

Species	Planting Location	Time of Planting	2018/2019	2019/2020
*Fagopyrum esculentum*	Belbies City Site	Mid-November	9.111de	9.444d
		Mid-January	9.334d	9.445d
		Mid-March	8.222ef	8.222e
	Sadat City Site	Mid-November	7.333 fg	7.778e
		Mid-January	7.667f	7.778e
		Mid-March	6.556 g	6.333f
*Fagopyrum tataricum*	Belbies City Site	Mid-November	14.667a	12.556a
		Mid-January	13.889a	12.111a
		Mid-March	13.778a	12.111a
	Sadat City Site	Mid-November	12.000b	11.333b
		Mid-January	10.667c	10.667c
		Mid-March	10.445c	10.667c
		LSD 0.05	**1.0341**	**0.6091**

### 3.5. Number of leaves per plant

**Fig 3:**
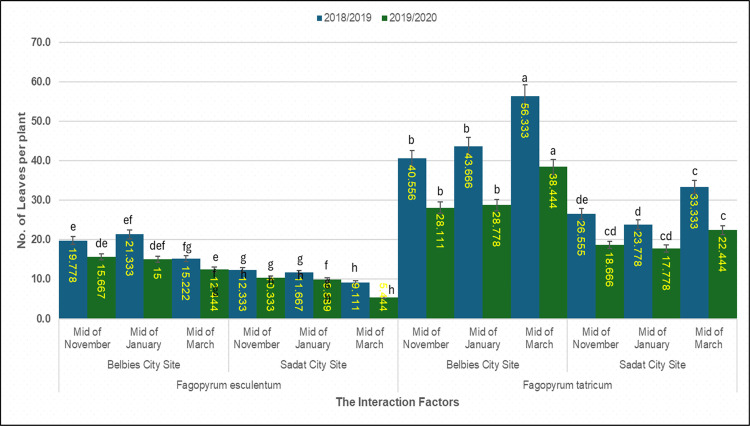
Branch number per plant of common and Tartary buckwheat across sowing dated and locations. Mean number of branches per plant of *Fagopyrum esculentum* and *Fagopyrum tataricum* sown at mid-November, mid-January and mid-March in Belbies city and Sadat city in 2018/19 (orange) and 2019/20 (blue); bars represnt the mean ±SE of three replicates, and different letters represent significant differences among sowing-date × location treatments within each species and season (one-way ANOVA with LSD post hoc test, p<0.05).

The three‐way factorial ANOVA indicated highly significant effects of species, location, sowing date, and their interactions on leaf number (p < 0.001; Table 6). At the Belbies city site, *F. tataricum* sown in mid-November presented the greatest canopy density, averaging 56.33 ± 1.12 leaves in 2018/19 and 38.44 ± 0.95 leaves in 2019/20. Leaf counts were lower **in** mid-March (40.56 ± 1.05 and 28.11 ± 0.88) than in mid-November. In comparison, *F. esculentum* at the same site formed significantly fewer leaves, with mid-November sowing yielding 19.78 ± 0.97 leaves in 2018/19 and 15.67 ± 0.85 in 2019/20, and 15.22 under mid-March sowing and 12.44 ± 0.72 under late sowing. In Sadat city, the number of leaves was uniformly lower: *F. tataricum* ranged from 33.33 ± 1.00 (mid-November) to 26.56 ± 0.92 leaves (mid-March) in 2018/19 and from 22.44 ± 0.78 to 18.67 ± 0.65 in 2019/20, whereas *F. esculentum* produced only 11.67 ± 0.85 to 9.11 ± 0.76 leaves (2018/19) and 9.89 ± 0.68 to 5.44 ± 0.58 leaves (2019/20) across sowing dates. The least significant difference (LSD) at p = 0.05 was 2.31 leaves in the first season and 2.05 leaves in the second season, confirming that the species, site, and planting time jointly and significantly governed foliar development (Fig 3).

**Table 6:** Number of leaves per plant by the interaction of species × location × sowing date.

Species	Planting Location	Time of Planting	2018/2019	2019/2020
*Fagopyrum esculentum*	Belbies City Site	Mid-November	19.778ef	15.667de
		Mid-January	21.333e	15.000def
		Mid-March	15.222 fg	12.444efg
	Sadat City Site	Mid-November	11.667gh	9.889 fgh
		Mid-January	12.333gh	10.333gh
		Mid-March	9.111 h	5.444 h
*Fagopyrum tataricum*	Belbies City Site	Mid-November	56.333a	38.444a
		Mid-January	43.666b	28.778b
		Mid-March	40.556b	28.111b
	Sadat City Site	Mid-November	33.333c	22.444c
		Mid-January	23.778de	17.778 cd
		Mid-March	26.555d	18.666 cd
		LSD 0.05	**4.5774**	**4.9744**

### 3.6. Fresh biomass per plant

**Fig 4:**
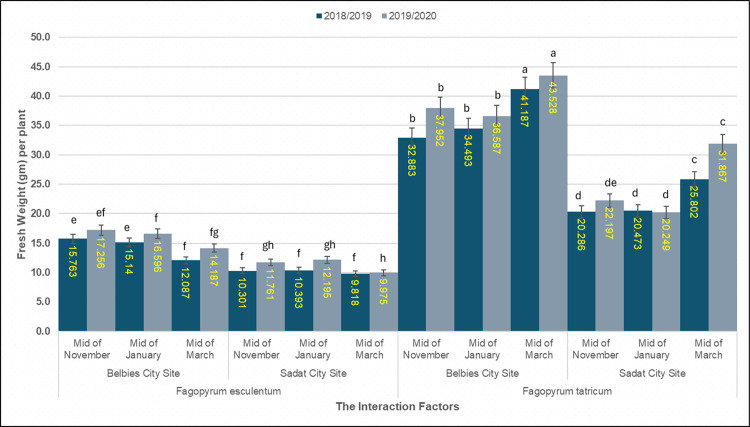
Internode count per plant of common and Tartary buckwheat across sowing treatments. Mean number of Internodes per plant of *Fagopyrum esculentum* and *Fagopyrum tataricum* sown at mid-November, mid-January and mid-March in Belbies city and Sadat city in 2018/19 (dark blue) and 2019/20 (orange); bars represnt the mean ±SE of three replicates, and different letters denote significant differences among sowing-date × location treatments within each species and season (one-way ANOVA with LSD post hoc test, p<0.05).

A significant species × location × sowing date interaction was detected for fresh biomass per plant (p < 0.001; Table 7). At the Belbies city site (BCS), *F. tataricum* sown in mid-November accumulated the greatest amount of biomass, averaging 41.19 ± 0.76 g in 2018/19 and 43.53 ± 0.81 g in 2019/20. *F. tataricum* biomass was 20% lower under mid-March sowing than under mid-November sowing (to 32.88 ± 0.69 g) and 13% lower (to 37.95 ± 0.74 g) in the two seasons. In contrast, *F. esculentum* at BCS reached only 15.76 ± 0.52 g (2018/19) and 17.26 ± 0.58 g (2019/20) under mid-November sowing—58–64% less than *F. tataricum*—and was lower under mid-March sowing (12.09–14.19 g). At the Sadat city site (SCS), the biomass of *F. tataricum* was markedly lower, ranging from 25.80 ± 0.61 g (mid-November) to 20.29 ± 0.57 g (mid-March), which was 38–51% lower than that at the BCS. *F. esculentum* at the SCS produced only 10.39–12.20 g, a 23–38% decrease compared with its performance at the BCS. The LSD at p = 0.05 was 2.04 g for 2018/19 and 2.18 g for 2019/20, underscoring the robustness of these differences across species, environments, and sowing dates (Fig 4).

**Table 7:** Fresh weight per plant by the interaction of species × location × sowing date.

Species	Planting Location	Time of Planting	2018/2019	2019/2020
*Fagopyrum esculentum*	Belbies City Site	Mid-November	15.763e	17.256ef
		Mid-January	15.140e	16.596f
		Mid-March	12.087f	14.187 fg
	Sadat City Site	Mid-November	10.393f	12.195gh
		Mid-January	10.301f	11.761gh
		Mid-March	9.818f	9.975 h
*Fagopyrum tataricum*	Belbies City Site	Mid-November	41.187a	43.528a
		Mid-January	34.493b	36.587b
		Mid-March	32.883b	37.952b
	Sadat City Site	Mid-November	25.802c	31.867c
		Mid-January	20.473d	20.249de
		Mid-March	20.286d	22.197d
		LSD 0.05	**2.7960**	**3.2579**

### 3.7. Number of seeds per plant

**Fig 5:**
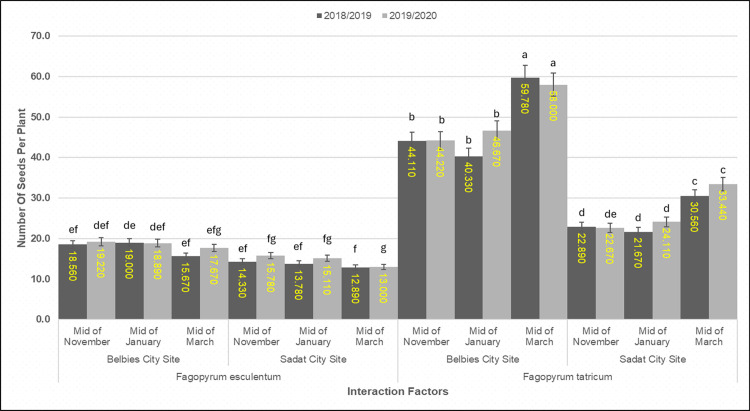
Leaf count per plant of common and Tartary buckwheat across sowing dated and locations. Mean number of leaves per plant of *Fagopyrum esculentum* and *Fagopyrum tataricum* sown at mid-November, mid-January and mid-March in Belbies city and Sadat city in 2018/19 (dark blue) and 2019/20 (green); bars represnt the mean ±SE of three replicates, and different letters denote significant differences among sowing-date × location treatments within each species and season (one-way ANOVA with LSD post hoc test, p<0.05).

**Fig 6:**
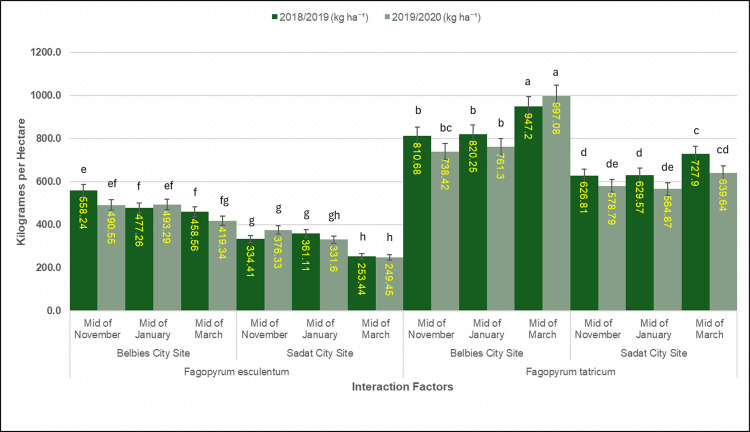
Fresh weight per plant of common and Tartary buckwheat across sowing dates and locations. Mean number of (g palnt ^˗1^) of *Fagopyrum esculentum* and *Fagopyrum tataricum* sown at mid-November, mid-January and mid-March in Belbies city and Sadat city in 2018/19 (dark blue) and 2019/20 (light blue); bars represnt the mean ±SE of three replicates, and different letters represnt significant differences among sowing-date × location treatments within each species and season (one-way ANOVA with LSD post hoc test, p<0.05).

Reproductive output displayed pronounced species × location × sowing date interactions (p < 0.001; LSD0.05 = 5.90 seeds in 2018/19, 5.38 in 2019/20). At the Belbies city site (BCS), *F. tataricum* sown in mid-March presented the highest seed set, averaging **59.78 ± 1.02** seeds per plant in 2018/19 and **58.00 ± 0.98** in 2019/20. In contrast, *F. esculentum* achieved only **18.56 ± 0.85** seeds per plant (2018/19) and **19.22 ± 0.88** (2019/20) under its optimal BCS in mid-November sowing—a 68–69% reduction relative to *F. tataricum*. At the Sadat city site (SCS), the number of seeds was approximately 40–50% lower for both species than at BCS.*F. tataricum* set **30.56 ± 1.10** (2018/19) to **33.44 ± 1.05** (2019/20) seeds per plant under mid-March sowing, whereas *F. esculentum* fell to **12.89 ± 0.72**–**14.33 ± 0.78** seeds per plant (Fig 5). The seed set was 15–25% lower under mid-March sowing than under mid-November sowing (Fig 6) (Table 8).

**Table 8:** Number of seeds per plant by the interaction of species × location × sowing date.

Species	Planting Location	Date of Planting	2018/2019	2019/2020
*Fagopyrum esculentum*	Belbies City Site	Mid-November	18.556def	19.222def
		Mid-January	19.000de	18.889def
		Mid-March	15.667ef	17.667efg
	Sadat City Site	Mid-November	14.333ef	15.778 fg
		Mid-January	13.778ef	15.111 fg
		Mid-March	12.889f	13.000 g
*Fagopyrum tataricum*	Belbies City Site	Mid-November	44.111b	44.222b
		Mid-January	40.333b	46.667b
		Mid-March	59.778a	58.000a
	Sadat City Site	Mid-November	22.889d	22.667de
		Mid-January	21.667d	24.111d
		Mid-March	30.555c	33.444c
	LSD 0.05		**5.9010**	**5.3804**

### 3.8. Grain yield (kg ha ⁻ ¹)

The grain yield mirrored the trends observed in vegetative vigor and seed set, with highly significant species × location × sowing date interactions (p < 0.001; LSD₀.₀₅: 68.8 kg ha ⁻ ¹ (2018/19) and 104.2 kg ha ⁻ ¹ (2019/20)). At the Belbies city site (BCS), *F. tataricum* sown in mid-March achieved the highest yields of **947 ± 22 kg ha ⁻ ¹** (2018/19) and **997 ± 25 kg ha ⁻ ¹** (2019/20), 75–80% higher than those of *F. esculentum* at its mid-November peak (558 ± 19 and 491 ± 18 kg ha ⁻ ¹; see Table 9). At the Sadat city site (SCS), *F. tataricum* yields 728 ± 20 (2018/19) and 627 ± 18 kg ha ⁻ ¹ (2019/20), 23–34% lower than those at BCS, whereas *F. esculentum* yields are 376 ± 15–253 ± 12 kg ha ⁻ ¹ (33–55% lower than those at BCS), the harsher sandy–loamy conditions.

**Table 9:** Grain yield (kg ha ⁻ ¹) by the interaction of species × location × sowing date.

Species	Planting Location	Time of Planting	2018/2019 (kg ha ⁻ ¹)	2019/2020 (kg ha ⁻ ¹)
*Fagopyrum esculentum*	Belbies City Site	Mid-November	558.24e	490.55ef
		Mid-January	477.26f	493.29ef
		Mid-March	458.56f	419.34 fg
	Sadat City Site	Mid-November	334.41 g	376.33 g
		Mid-January	361.11 g	331.60gh
		Mid-March	253.44 h	249.45 h
*Fagopyrum tataricum*	Belbies City Site	Mid-November	810.68b	738.42bc
		Mid-January	820.25b	761.30b
		Mid-March	947.20a	997.08a
	Sadat City Site	Mid-November	626.81de	578.79de
		Mid-January	629.57d	564.87de
		Mid-March	727.90c	639.64 cd
		LSD 0.05	**68.786**	**104.17**

## 4. Discussion

### 4.1. Effect of species × location × sowing date on plant height (cm)

The three‐factor RCBD (Section 2.5) demonstrates that plant height in buckwheat is governed by a complex interplay of genotype, soil environment, and sowing date. However, Tartary buckwheat (*F. tataricum*) consistently outperformed common buckwheat (*F. esculentum*) in both experimental Belbies city and sandy-loamy (Sadat city) soils, reflecting the consistently greater field performance of *F. tataricum* at our two locations and sowing dates and its resilience to suboptimal conditions [26,73]. At Belbies—an irrigated Nile Delta site with relatively high organic matter (0.75%) and available P (5.25 mg kg ⁻ ¹)—mid-November sowing maximized canopy extension in F. tataricum (102.00 cm), with only an 8.**4%** reduction under late (mid-March) sowing, whereas F. esculentum **experienced** a 14.**5%** height loss (Table 3). These results align with established findings that early planting exploits cooler temperatures and adequate moisture to support meristematic activity [42,74].

**Fig 7:**
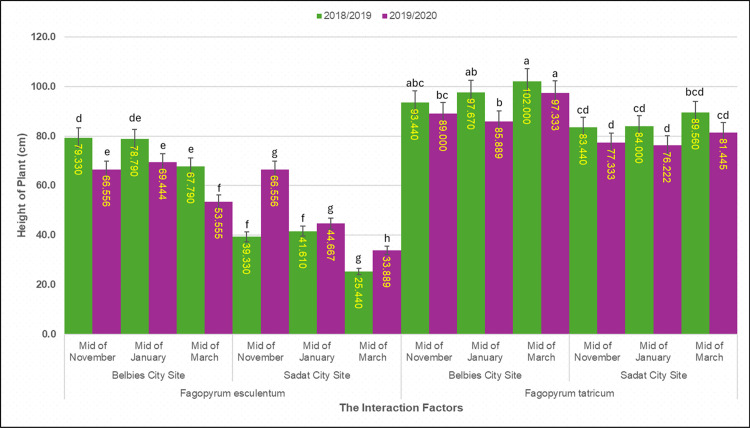
Number of seeds per plant of common and Tartary buckwheat across sowing dates and locations. Mean seed count per plant (±SE; n=10 plants ×3 replicates) for *Fagopyrum esculentum* and *Fagopyrum tataricum* sown at mid-November, mid-January and mid-March in Belbies city site (BCS) Sadat city site (SCS) in 2018/19 (dark bars) and 2019/20 (light bars) seasons. Yellow labels indicate mean values. Different lowercase letters above the bars denote significant differences among species× location × sowing-date treatments within each season (one-way ANOVA with LSD post hoc test, p<0.05).

In Sadat city—on the desert fringe with lower nutrient retention (OM 0.18%, P 1.7 mg kg ⁻ ¹)—both species presented reduced stature, but the proportional decline in *F. esculentum* (48–68%) was far greater than that in F. tataricum (11–18%), indicating that *F. tataricum* maintained taller plants than *F. esculentum* across sites and sowing dates. We did not measure antioxidant/osmoprotectant pathways in this study. [27,75]. The smaller height reduction under delayed sowing for F. tataricum (6–8%) than for *F. esculentum* (14–39%) further highlights the species-specific stability in height under relatively high temperature and limited moisture conditions during the reproductive stage [46]. Therefore, by situating both sites on a unified regional map (Fig 1) and applying identical compost and superphosphate regimes (Section 2.3), we confirm that the observed height differences primarily reflect the combined influence of soil texture, microclimate, and irrigation rather than management-related artifacts [35,76]. These findings emphasize the ecological complexity underlying growth responses and support the recommendation to prioritize *F. tataricum* and early sowing schedules to optimize vegetative growth in semiarid Egyptian systems (Fig 7).

### 4.2. Effect of species × location × sowing date on the number of branches per plant

**Fig 8:**
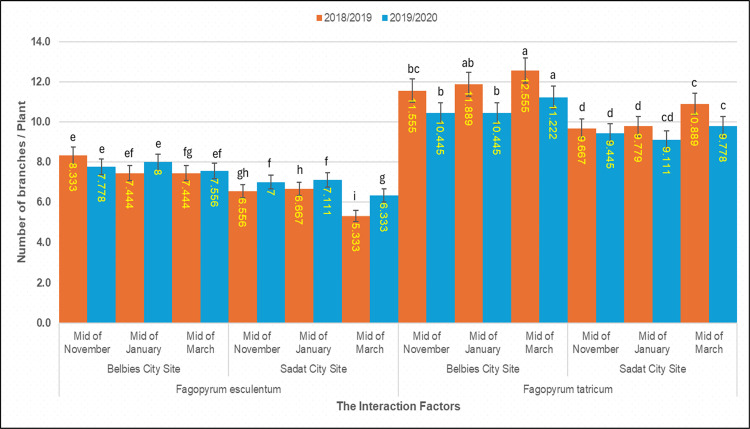
Grain yield of common and Tartary buckwheat across sowing dated and locations. Mean Grain yield (kg ka ^˗1^) of *Fagopyrum esculentum* and *Fagopyrum tataricum* sown at mid-November, mid-January and mid-March in Belbies city and Sadat city in 2018/19 (dark green) and 2019/20 (light green); bars show the mean ±SE of three replicates, and different letters denote significant differences among sowing-date × location treatments within each species and season (one-way ANOVA with LSD post hoc test, p<0.05).

Our data shows significant differences in branch number between the two buckwheat species (*Fagopyrum tataricum* and *Fagopyrum esculentum*), with variations influenced by location and sowing date across both seasons (Fig 8). However, *F. tataricum* consistently produced more branches per plant than did *F. esculentum*, regardless of location or sowing date. Branch numbers ranged from 9.67 (in Sadat city, mid-March 2018/2019) to 12.56 (in Belbies city, mid-November 2018/2019) for *Fagopyrum tataricum* and from 5.33 to 8.33 for *Fagopyrum esculentum* under the same conditions. These findings agree with previous studies suggesting that *F. tataricum* has a genetically determined advantage in branch development, which is likely linked to its greater vegetative vigor and efficient resource allocation [77,78,79].

The planting location significantly affected branch numbers in both species, with plants growing in Belbies city producing more branches than those growing in Sadat city. This pattern paralleled that observed for plant height and reflected the more favorable soil and environmental conditions at Belbies city—higher organic matter and nutrient availability supporting better root growth and branch initiation [35,76]. These favorable conditions facilitate increased nutrient uptake, improved water retention, and optimal root development, all of which are crucial for stimulating branch proliferation. In Sadat city, sandy-loamy soil with low nutrient retention and moisture capacity likely has limited branching due to reduced nutrient and water availability [39,28]. Furthermore, the timing of sowing significantly affected the number of branches produced by both species. In both seasons and locations, mid-November and mid-January sowing produced higher branch numbers than did mid-March sowing. Early sowing provides an extended vegetative growth period and favorable environmental conditions, enabling optimal resource allocation for branching [42,80,74]. Under mid-March sowing, the shorter vegetative phase and higher temperature are likely limited branch development [41,55,81]. The significant interactions among species, location, and sowing date underscore the combined influence of genetic and environmental factors on buckwheat branching. *F. tataricum* has relatively stable branch production across environments, reflecting its relatively broad adaptability to variable conditions [82,75].

### 4.3. Effect of Species × Location × Sowing Date on the Number of Internodes per Plant

**Fig 9:**
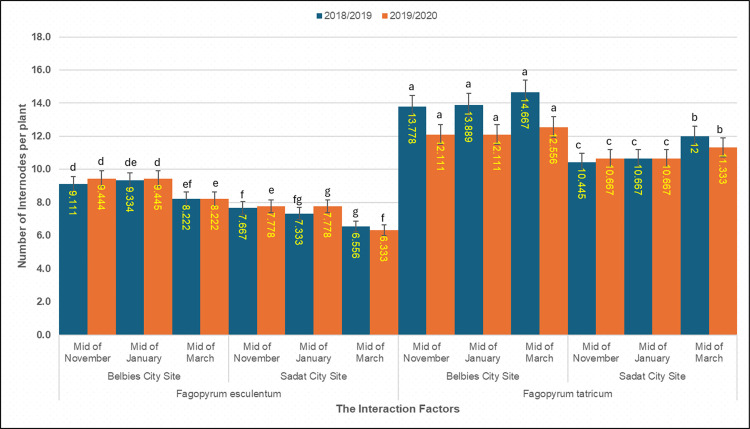
Comparative establishment of both cultivated buckwheat in Belbies city (BCS) and Sadat city (SCS). In Section A (panels “a” to “e”; for BCS), while plots amended one eek before sowing with 10t ha ˗1 compost and 150 kg ha ˗1 single superphospate were hand-seeded (After cultivation) and, by 14 days after sowing, presented uniform cotyledon emergence, precise row formation following first thinning, and a dense vegetative canopy under clay sandy conditions. In Section B (panels “f” to “j”; for the SCS), sandy–loamy beds were manually prepared and broadcast-seeded, with seedling emerging at 14 DAS under desert-fringe conditions an dproccessing through midscason row development to weel-defined vegetative satngs just befor reproductive transitions.

The number of internodes per plant showed clear species-, location-, and sowing date–dependent patterns, similar to those observed for plant height and branching (Fig 9). The species differences were pronounced; *F. tataricum* consistently produced more internodes (10.45–14.67) than did *F. esculentum* (6.56–9.44) across sites and sowing dates. This greater internode formation in *F. tataricum* likely reflects genetic differences influencing vegetative vigor and node initiation, as noted in previous studies [77,79]. Location also had a marked effect on internode number. Compared with those in the sandy-loamy soils of Sadat city, the plants in Belbies city, where the soils presented greater nutrient and moisture retention, presented more internodes of both species. For example, *F. tataricum* in mid-November averaged 14.67 internodes at Belbies versus 12.00 at Sadat city in 2018/2019. The relatively high soil fertility and water-holding capacity of Belbies likely support relatively strong apical meristem activity and node differentiation [35,76]. However, sowing date also significantly affected internode development. Early sowing (mid-November and mid-January) consistently yielded more internodes than late sowing did (mid-March) across both locations and seasons. Earlier sowing provides longer vegetative growth periods that favor node formation, whereas delayed sowing shortens this phase, reducing the internode number [42,74]. Therefore, the pronounced species × location × sowing date interaction highlights the combined influence of genetic potential and environmental conditions. *F. tataricum* maintained relatively stable internode numbers across sowing dates, particularly at Belbies, whereas *F. esculentum* showed greater reductions under later sowing and in less fertile soils. This stability suggests broader adaptability of *F. tataricum* to variable field environments [82,75].

### 4.4. Effect of species × location × sowing date on the number of leaves per plant

In Belbies city (clay‒sandy soil: OM 0.75%, P 5.25 mg kg ⁻ ¹), early-sown *F. tataricum* produced 56.33 leaves per plant—approximately three times the 19.78 leaves of *F. esculentum*—under identical compost (10 t ha ⁻ ¹) and superphosphate (150 kg ha ⁻ ¹ P₂O₅) applications (Section 2.3) [77,79]. These differences reflect the combined influence of Belbies’ greater nutrient and moisture retention and the species-specific growth potential of *F. tataricum* rather than the fertilizer regime alone [35,76].

At the sandy-loamy Sadat site (OM 0.18%, P 1.7 mg kg ⁻ ¹; Fig 1), the leaf counts for both species were lower, but *F. tataricum* maintained 33.33 leaves per plant compared with 11.67 for *F. esculentum*, confirming its consistently greater leaf number across sites and highlighting that *F. tataricum* sustained greater leaf numbers than did *F. esculentum* at both sites. References to FtATGs and NF-Y derive from prior literature and were not tested here. However, delaying sowing to mid-March resulted in 20–30% lower leaf numbers during mid-March sowing than during mid-November sowing (to 40.56 and 15.22 leaves for Tartary and common buckwheat, respectively) because of a shortened vegetative window and exposure to higher temperatures (>30 °C) and lower soil moisture at critical growth stages (Table 10) [42,83]. Notably, a smaller proportional decline indicates a relatively stable biomass of *F. tataricum* under the two site contexts and sowing windows evaluated [74,75]. The highly significant species × location × sowing date interaction (p < 0.05, LSD) indicates that maximizing canopy development—and thus photosynthetic potential and yield—depends on combining *F. tataricum* with fertile, irrigated sites such as Belbies and maintaining early sowing schedules (mid-November). This integrated interpretation links experimental design (Section 2.5), consistent management (Section 2.3), and site context (Fig 1) to the observed vegetative responses (Fig 3).

**Table 10:** Monthly weather parameters, including temperature, precipitation, relative humidity, and soil conditions, at the Belbies city site (BCS) and the Sadat city site (SCS) during the 2018–2019 and 2019–2020 growing seasons.

Location	(1) Belbies City Site (BCS) = 30.4196° N, 31.5619° E																				
unit	°C				mm	%					cm	km/h					min	°C			
level	2 m elevation corrected				sfc	2 m					sfc	sfc					sfc	0-7 cm down			
aggregation	Max	Min	Mean		Summ ation	Max	Min		Mean		Summation	Max	Min		Mean		Summation	Max	Min		Mean
timestamp	Temperature [2 m elevation corrected]				Precip Total	Relative Humidity [2 m]					Snowfall Amount	Wind Gust					Sunshine Duration	Soil Temperature [0-7 cm down]			
**1**^**st**^ **Season (2018/2019)**																					
**2018-11**	27.31	15.78	21.07		0.20	84.88	33.30		59.32		0.00	27.53	8.10		17.41		492.23	28.01	19.28		23.25
**2018-12**	21.80	12.64	16.76		0.10	77.40	38.65		57.65		0.00	35.29	12.45		23.07		365.42	22.14	15.40		18.55
**2019-01**	19.66	8.51	13.65		0.00	68.30	29.66		48.53		0.00	40.62	14.46		26.20		513.44	19.87	11.27		15.22
**2019-02**	21.53	9.61	15.25		0.47	85.94	30.03		56.60		0.00	37.14	11.30		23.52		443.71	21.21	12.60		16.73
**2019-03**	23.60	12.02	17.43		0.22	83.98	29.14		55.71		0.00	39.86	10.34		25.65		490.35	24.48	15.49		19.50
**2019-04**	28.02	14.49	20.98		0.15	78.60	22.58		49.06		0.00	39.10	11.45		25.22		612.82	29.81	18.42		23.60
**2019-05**	35.09	19.20	26.76		0.00	74.37	19.63		42.99		0.00	38.97	11.82		25.67		748.58	36.57	23.43		29.56
**2019-06**	36.38	23.41	29.60		0.00	80.81	27.78		51.95		0.00	38.34	9.44		24.10		764.44	39.03	27.63		32.93
**2019-07**	36.71	24.48	30.37		0.00	80.71	29.94		54.06		0.00	32.50	8.22		20.93		777.84	40.08	28.89		34.03
**2019-08**	36.40	24.31	30.15		0.00	83.86	31.48		57.11		0.00	29.52	7.72		19.12		731.51	39.27	28.59		33.46
**2**^**nd**^ **Season (2019/2020)**																					
**2019-11**	28.57	16.53	21.96		0.00	80.22	32.05		56.07		0.00	30.60	10.32		19.44		565.12	28.89	19.82		23.98
**2019-12**	22.24	11.96	16.67		0.22	76.33	36.13		56.52		0.00	38.10	12.58		24.13		453.97	22.38	14.95		18.36
**2020-01**	19.04	9.75	14.18		0.09	77.87	38.51		57.70		0.00	39.40	14.24		26.27		413.80	19.70	12.57		15.90
**2020-02**	21.39	10.79	15.77		0.16	81.57	35.56		58.49		0.00	35.71	9.19		22.01		421.16	21.77	13.64		17.39
**2020-03**	24.75	12.81	18.46		1.42	81.77	31.91		55.69		0.00	40.18	12.27		25.93		540.14	24.92	16.33		20.21
**2020-04**	28.27	15.43	21.59		0.06	80.38	26.19		51.30		0.00	37.34	10.33		23.96		631.75	30.08	19.56		24.33
**2020-05**	33.92	18.94	26.05		0.02	75.52	22.22		46.28		0.00	38.90	11.33		24.83		754.07	35.83	23.54		29.15
**2020-06**	35.80	21.27	28.24		0.00	79.10	24.79		48.35		0.00	37.56	11.08		23.93		804.67	38.58	26.29		31.85
**Location**	**(2) Sadat City Site (30.41°N 30.53°E) – Experimental Farm of the University of Sadat City**																				
**1**^**st**^ **Season (2018/2019)**																					
**2018-11**	26.07	15.28		20.24	0.13	86.93		37.89		63.71	0.00	28.36		9.79		18.44	514.65	28.94	17.16	22.25	
**2018-12**	20.53	12.11		15.95	0.11	81.35		44.88		63.61	0.00	35.08		13.78		24.05	367.36	22.14	13.37	17.22	
**2019-01**	18.37	7.97		12.92	0.00	70.88		33.01		52.09	0.00	40.59		15.38		27.21	528.03	20.32	9.00	13.98	
**2019-02**	20.27	9.20		14.54	0.06	86.24		32.95		58.55	0.00	37.88		12.66		24.44	476.37	22.67	10.38	15.92	
**2019-03**	22.46	11.51		16.64	0.12	86.99		32.94		60.15	0.00	40.58		11.68		26.14	519.65	26.08	13.21	18.80	
**2019-04**	26.73	13.95		20.05	0.07	81.27		26.37		52.91	0.00	39.68		12.11		25.61	659.17	31.91	16.01	23.06	
**2019-05**	33.74	18.70		25.92	0.00	75.50		21.26		45.37	0.00	39.11		11.96		25.40	759.04	39.26	21.10	29.36	
**2019-06**	34.96	22.70		28.34	0.00	86.65		30.68		57.62	0.00	41.82		11.76		26.38	771.71	41.50	25.27	32.50	
**2019-07**	35.65	23.73		29.33	0.00	87.23		31.69		59.56	0.00	39.69		10.93		24.42	788.43	43.21	26.55	33.93	
**2019-08**	35.86	23.87		29.33	0.00	89.44		31.53		61.52	0.00	37.06		10.06		22.81	724.38	43.27	26.96	33.97	
**2**^**nd**^ **Season (2019/2020)**																					
**2019-11**	27.71	16.05		21.55	0.02	83.03		33.72		59.30	0.00	30.70		11.03		19.84	566.58	30.35	17.62	23.26	
**2019-12**	21.16	11.92		16.25	0.21	79.32		41.03		60.84	0.00	38.00		13.61		25.39	479.10	22.91	13.20	17.48	
**2020-01**	17.90	9.57		13.61	0.08	80.72		42.92		62.32	0.00	39.73		15.86		27.51	402.65	20.31	10.87	15.02	
**2020-02**	19.83	10.56		14.96	0.30	83.40		41.75		64.11	0.00	36.11		9.88		23.03	431.64	22.33	11.89	16.41	
**2020-03**	23.52	12.48		17.71	1.12	82.22		35.36		58.70	0.00	40.53		12.38		26.23	554.82	26.13	14.21	19.35	
**2020-04**	26.71	14.74		20.43	0.00	83.89		28.41		55.47	0.00	38.86		11.32		24.71	629.39	32.63	17.13	23.86	
**2020-05**	32.48	18.27		24.98	0.00	79.23		24.26		49.79	0.00	41.12		12.11		26.33	760.11	38.00	20.78	28.52	
**2020-06**	33.92	20.64		26.81	0.00	84.67		27.79		54.32	0.00	40.31		12.20		25.33	802.50	40.71	23.56	31.14	

### 4.5. Effect of species × location × sowing date on fresh biomass per plant

The fresh weight per plant was highly significantly affected by the species × location × sowing date interaction (p < 0.05, LSD). Species effects were dominant: at Belbies city, the mid-November sowing of *F. tataricum* yielded 41.19 g and 43.53 g fresh weight in 2018/19 and 2019/20, respectively—over 2.6 times greater than that of *F. esculentum* (15.76 g and 17.26 g) under the same conditions, indicating that the higher biomass accumulation potential of this species was driven by increased meristem activity and photosynthetic efficiency [77,79]. However, location effects further modulated these species differences. Belbies’ clay‒sandy soil (OM 0.75%, P 5.25 mg kg ⁻ ¹) presented substantially higher fresh weights than did Sadat’s sandy-loamy soil (OM 0.18%, P 1.7 mg kg ⁻ ¹), where *the* fresh weight of F. tataricum averaged 25.80 g and 31.87 g and that of *F. esculentum* ranged from 10.39 g and 12.20 g. These findings underscore the importance of the soil water-holding capacity and nutrient availability—optimized by our standardized amendment regime—in sustaining biomass production, with poorer retention at Sadat limiting both species [35,76]. Sowing date effects were equally pronounced. The fresh weight of *F. tataricum* (to 32.88 g and 37.95 g) was 20–25% lower than that of *F. esculentum* (to 12.09 g and 14.19 g) at mid-March sowing and 23–30% lower than that of F. esculentum at mid-November sowing. Although leaf numbers were lower at mid-March sowing, the fresh biomass per plant was greater in *F. tataricum* because individual leaves and stems were thicker and more succulent, leading to a higher water content per unit of tissue. Warmer conditions promoted rapid cell expansion and increased specific leaf weight (SLW), whereas early-sowing plants developed more but thinner leaves. This morphological compensation explains how the total fresh weight increased despite fewer leaves, reflecting a shift from extensive to intensive leaf growth under elevated temperatures [82,75,73]. The smaller proportional decline in *F. tataricum* suggests greater stability of biomass production under varying conditions, which is consistent with earlier reports on FtATG-related metabolic regulation [27,73]. Thus, the significant species × location × sowing date interaction indicates that biomass was greatest for *F. tataricum* at Belbies and for earlier sowings in our trials; extrapolation to other sites or managements requires further testing (mid-November) as per our results in Fig 4. These findings demonstrate how genetic, edaphic, and phenological factors jointly determine buckwheat productivity in Egyptian agroecosystems [60,61].

### 4.6. Effect of species × location × sowing date on the number of seeds per plant

Tartary buckwheat presented a clear advantage in terms of seed set, which was associated with greater floret fertility and more efficient partitioning of assimilates to reproductive organs. Under optimal conditions in Belbies city, *F. tataricum* has nearly 60 seeds per plant—over three times the output of *F. esculentum*—a pattern consistent with that of Aubert et al. [26] and Cheng et al.  [84] who reported superior floret initiation and retention in *F. tataricum* populations under various environmental stresses. Previous studies have shown that increased expression of *FtATG* genes in *F. tataricum* is associated with increased antioxidant activity and may help maintain phloem function and ovule viability during grain filling [73]. However, the approximately 40–50% reduction in seeds per plant at the sandy-loamy Sadat site underscores the limiting role of soil physical properties in reproductive success. Our results mirror those of Zhou et al. [76], who reported that the low water and nutrient-holding capacity of sandy soils limits grain filling in buckwheat, even under uniform amendment. Clay‒sandy soils such as those at Belbies retain more moisture and nutrients, supporting threefold greater seed set and nutrient availability and promoting threefold greater seed set, which is in agreement with the findings of Phalempin et al. [35]. Seed numbers were 15–25% lower under mid-March sowing than under mid-November sowing for both species, highlighting the critical impact of phenological synchrony with favorable environmental windows. This trend aligns with Jung et al. [42] and Zhang et al.  [85], who reported that late-season high temperatures accelerate senescence and shorten the grain-filling period in buckwheat, reducing the final seed number. Erol et al. [45] and Zhang et al. [85] similarly reported up to 30% seed loss with delayed planting under semiarid conditions. Crucially, *F. tataricum* presented a relatively small decline in seed set under both edaphic stress and delayed sowing, demonstrating its reproductive stability. These patterns are consistent with prior reports that stress-responsive pathways in *F. tataricum* help maintain floral function under heat and drought [30,31]. This stability supports the use of *F. tataricum* in various agroecosystems in Egypt and its potential role in breeding for climate adaptation.

The markedly lower grain yield of *F. esculentum* than that of *F. tataricum* can be attributed in part to pollination biology. *F. esculentum* is largely self-incompatible and depends on insect pollinators—particularly honeybees (*Apis mellifera*) and wild bees—for effective fertilization [86,87]. During both growing seasons, no managed bee hives were located near either experimental site, and natural pollinator activity was sparse, especially in Sadat city, where the vegetation cover is low. In contrast, *F. tataricum* is predominantly self-fertile and can set seeds without pollinators. This difference in the reproductive system, combined with the drier microenvironment of the desert fringe site, explains the very low seed set and yield observed for *F. esculentum* [75,76].

### 4.7. Effect of species × location × sowing date on grain yield (kg ha⁻¹)

The interactions among species, location, and sowing date revealed distinct adaptive differences between *F. esculentum* and *F. tataricum* across Egypt’s contrasting agroecosystems. Across both seasons, *F. tataricum* produced higher yields than did *F. esculentum* at all locations and sowing dates, although the extent of its advantage depended on the site and planting time. In Belbies city (clay‒sandy, well-irrigated Nile Delta), in Belbies city, the mid-March sowing of *F. tataricum* produced the highest yields—947 and 997 kg ha ⁻ ¹ in 2018/19 and 2019/20—while in Sadat city (sandy-loamy, desert fringe), yields were 728 and 640 kg ha ⁻ ¹, respectively. This >200 kg ha ⁻ ¹ loss at Sadat reflects the combined effects of lower soil water‐holding capacity (Table 1) and higher evapotranspiration under its warmer, windier microclimate (Table 10), illustrating that *F. tataricum* yielded more than *F. esculentum* did at both sites, although absolute yields were lower at the sandy–loamy desert-fringe site [27,73]. In contrast, *F. esculentum* yielded less than 560 kg ha ⁻ ¹, with its highest values (558 and 491 kg ha ⁻ ¹ in 2018/19 and 2019/20) recorded at Belbies under mid-November sowing. The yields of *F. esculentum* are 253–334 kg ha ⁻ ¹ under mid-March sowing at Sadat, indicating a steeper decline with delayed planting and greater sensitivity to shorter vegetative periods and moisture stress [42,83]. Uniform agronomic management (compost + TSP application, irrigation schedule) and two‐step ANOVA confirm that these patterns represent true species × environment × time interactions rather than plot variability [41]. Importantly, while Belbies’ clay‐sandy soils and reliable irrigation supported higher yields overall, the ranking of treatments remained consistent across sites (*F. tataricum* mid‒March > *F. tataricum* mid‒January/November > *F. esculentum* mid‒November > later dates), emphasizing that species and sowing date had stronger influences than soil texture alone [88,89]. These results indicate that mid-March sowing of *F. tataricum* optimizes biomass accumulation and grain filling by aligning key growth stages with moderate spring temperatures and residual soil moisture [49,50], whereas *F. esculentum* performs better under earlier planting to avoid late-season heat. Moreover, *F. esculentum* may be better suited to earlier planting windows to avoid late‐season heat stress. This understanding of species × environment × time interactions provides a basis for developing species-specific sowing schedules adapted to Egyptian agroecosystems.

## Conclusions

Three-factor field trials demonstrated that *Fagopyrum tataricum* consistently produced greater vegetative growth, reproductive output, and grain yield than did *Fagopyrum esculentum* across two contrasting Egyptian agroecosystems and three sowing dates. The significant species × location × sowing date interactions highlight that optimizing buckwheat performance requires matching species selection with the soil environment and planting time rather than treating these factors independently. Early sowing (mid-November) favored canopy development and biomass accumulation, whereas later sowing (mid-March) synchronized reproductive stages with residual soil moisture and moderate temperatures, particularly benefiting *F. tataricum* at the clay–sandy Nile Delta site. In the sandy–loamy desert fringe, across both locations and the three sowing dates evaluated, *F. tataricum* exceeded *F. esculentum* in vegetative traits, seed set, and grain yield. These field comparisons support *F. tataricum* as a strong candidate for Egyptian agroecosystems, with the highest yields observed at Belbies under mid-March sowing. Controlled studies are needed to test specific drought/heat or nutrient-stress tolerance mechanisms.

Integrating *Tartary buckwheat* into Egypt’s crop portfolio could enhance diversification, nutritional security, and climate resilience to increase diversification, nutritional security, and climate resilience. Future research should isolate the effects of individual soil amendments, evaluate irrigation regimes under water-limited scenarios, and use molecular and physiological analyses to clarify the mechanisms underlying *Tartary buckwheat* performance.

## Supporting information

S1 Fig Graphical abstract buckheat 3 way. (PDF)

S1 Data Supplementary tables. (XLSX)
