# Supplementary figures and images for: The impact of environmental factors, sowing time, and soil conditions on the growth, yield, and resilience of Fagopyrum tataricum and Fagopyrum esculentum in Egyptian agroecosystems
Source: PLoS One. 2026 May 14;21(5):e0344039. doi: 10.1371/journal.pone.0344039 (PMC13175345; doi:10.1371/journal.pone.0344039)

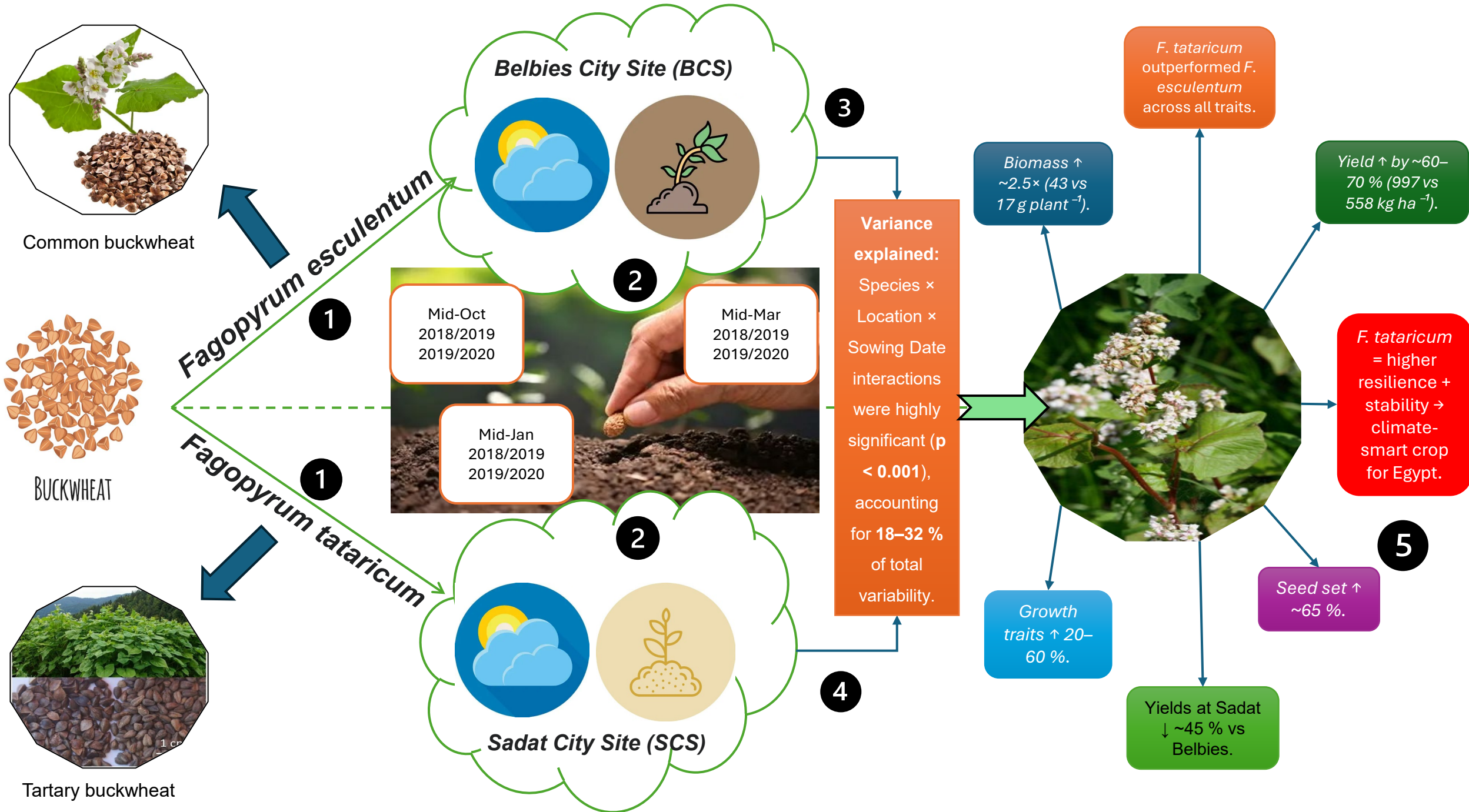

Supplement: S1 Fig — (PDF) [file pone.0344039.s001.pdf]
